# Application of Traditional Chinese Medicine in Treatment of Atrial Fibrillation

**DOI:** 10.1155/2017/1381732

**Published:** 2017-01-24

**Authors:** Yan Dong, Jiangquan Liao, Kuiwu Yao, Wenrui Jiang, Jie Wang

**Affiliations:** ^1^Department of Cardiology, Guang'anmen Hospital, China Academy of Chinese Medical Sciences, Beijing, China; ^2^Graduate School, Beijing University of Chinese Medicine, Beijing, China

## Abstract

Atrial fibrillation (AF) is the most common cardiac arrhythmia, which is related to many cardiac and cerebral vascular diseases, especially stroke. It can therefore increase cardiovascular mortality and all-cause death. The current treatments of AF remain to be western drugs and radiofrequency ablation which are limited by the tolerance of patients, adverse side effects, and high recurrence rate, especially for the elderly. On the contrary, traditional Chinese medicine (TCM) with long history of use involves various treatment methods, including Chinese herbal medicines (CHMs) or bioactive ingredients, Chinese patent medicines, acupuncture, Qigong, and Tai Chi Chuan. With more and more researches reported, the active roles of TCM in AF management have been discovered. Then it is likely that TCM would be effective preventive means and valuable additional remedy for AF. The potential mechanisms further found by numerous experimental studies showed the distinct characteristics of TCM. Some CHMs or bioactive ingredients are atrial-selective, while others are multichannel and multifunctional. Therefore, in this review we summarized the treatment strategies reported in TCM, with the purpose of providing novel ideas and directions for AF management.

## 1. Introduction 

Atrial fibrillation (AF) is the most common cardiac arrhythmia which will continue to grow rapidly. The estimated lifetime risk of developing AF is 25% in people over 40 years [1]. Another investigation [2] showed that the elderly had higher prevalence than the young. Current four principles of AF treatment include rate control, rhythm control, antithrombotic therapy, and dealing with the primary disease (or risk factors). So far western medicines remain the major treatment strategy of rate and rhythm control. Their positive actions are definite in emergency situations but still limited by recurrence of AF, visceral injuries, and inevitable adverse effects, such as severe ventricular arrhythmia [3], especially for long-time use. Another effective option is catheter ablation, which however has high recurrence and patients often have to receive once more operations. So this is one bottleneck met in the treatment of AF. The other one neck of bottle occurs in antithrombotic therapy which contains anticoagulation, antiplatelet aggregation, and fibrinolytic in general. All of them have high risk of bleeding, especially for the old persons. Even if antithrombotic therapy is the key of AF treatment, many patients have not received enough treatment or have been in poor INR control [4, 5], owing to the factors of both doctors and the patients [6]. Thereby in spite of some progress made in AF management, there still exists many critical problems.

On the contrary, traditional Chinese medicine (TCM), as complementary and alternative treatment, therefore becomes a viable option for these AF patients. It is reported that some single Chinese herbal medicines (CHMs) or bioactive ingredients, traditional Chinese patent medicines, and nondrug methods including acupuncture, Qigong, and Tai Chi Chuan can play active roles in the four principles of AF treatment, respectively, with low adverse reaction. What is more, they can lower the degree of discomforts like palpitation, chest discomfort, and dyspnea and then increase the tolerance of patients to disease, thus obviously improving their life quality. Further, multichannels are reportedly involved in the action mechanisms, including regulation of ion channels [7, 8], inhibition of inflammatory factors [9], activity of antioxidant [10, 11], and effect of antiplatelet aggregation [12]. Additionally, some CHMs or bioactive ingredients selectively work on the atrial myocytes, thus avoiding the property of proarrhythmia. In this review, we summarized these distinct types of TCMs with the purpose of demonstrating the alternative treatment of AF in perspective of TCM.

## 2. Single CHMs or Bioactive Ingredients

CHMs have long history of use basis with the theory of TCM in China. A former research showed around 13,000 herbs were estimated to be usually used, and more than 100,000 medicinal recipes had been recorded in China [13]. These data are bound to be updated since the wider acceptance and usage now than before. The effectiveness and safety of these herbs are confirmed by more and more researches, and potential mechanisms are gradually being discovered. AF, as a common refractory disease, has attracted people over the world to study therapeutic Chinese herbs targeting itself, which in return highlights the advantages of CHMs.

### 2.1. *Berberis vulgaris* L. 

*Berberis vulgaris* L. (1753) is the most well-known species of* Berberis* that belongs to family Berberidaceae and evergreen shrubs throughout the temperate and subtropical regions of the world (apart from Australia). Its berries were used for culinary purposes in ancient Europe and Iran, and the flowers had the function of treating musculoskeletal pain in the theory of TCM for many centuries while current researches have focused on the extract from barberry root, berberine, which belongs to isoquinoline alkaloid. Berberine is considered to be a potential agent for AF because of its effects on primary disease, rate control, and rhythm control. It is reported to have these medicinal properties of vasodilation, positive ionotropic, and negative chronotropic actions [7], by which some primary diseases of AF like coronary heart disease and heart failure can be relieved. Further, it can suppress acetylcholine-induced AF in the rabbit through increasing effective refractory period (ERP) and prolonging the action potential duration (APD) of atrial myocytes [14]. This function was demonstrated to be in a dose-dependent manner [7] and in the biochemical mechanism of inhibiting the integral of transient outward potassium current (Ito) [7]. Moreover, berberine is a typical multichannel ion blocker. Previous studies have showed that it can inhibit KATP [15], IKV [16], IKCa [16], IK1, and IK [17]. Regrettably, though berberine shows promising function on AF (Table 1), the advantages have not been systematically studied in human clinical trials. And we should use it with caution, for it is demonstrated to be a potent inhibitor of CYP3A4 enzyme which involves in the metabolism of many medications [18].

### 2.2. *Saussurea involucrata*

*Saussurea involucrata* (Kar. & Kir.) Sch. Bip. (1846), named snow lotus in central Asia, is a genus of flowering plants in the thistle tribe within the family Asteraceae. It is perennial herbaceous plant and precious medicinal material, with the treatment of musculoskeletal pain and tonifying kidney effect in ancient China.* Saussurea* flowers and stems are therefore widely used in rheumatic disease. Recent studies further illustrated that* Saussurea* and the sesquiterpene lactone fraction of* Saussurea lappa* roots had anti-inflammatory and analgesic effects [19–21]. In addition, the flavone compound of* Saussurea*, acacetin, was showed to prolong APD and ERP without prolonging the corrected QT interval in atrial myocytes [8], thus suppressing AF in dogs. The potential mechanism was the inhibition of acetylcholine-activated potassium current (Kach), ultrarapid delayed rectifier (IKur), and transient outward (Ito) potassium current as an atrial-selective agent [8]. Compared with the TCM theory, acacetin is a new discovery which is an effective and promising choice for AF (Table 1), but limited to animal experiments. Moreover, researchers failed to discover the relationship between anti-inflammatory and restraint myocardial remodeling, which may provide new direction for preventing the occurrence and development of AF. Therefore, further studies are needed to excavate the potential mechanism and relevant clinical investigations are urgently required.

### 2.3. Crataegus rhipidophylla Gand

Crataegus rhipidophylla Gand. (1872) is also called hawthorn that belongs to a large genus of shrubs and trees in the family Rosaceae. It is believed to promote blood to run around the whole body in the TCM theory. Based on this theory, a research discovered that two proanthocyanidins of its flower heads, catechin and epicatechin, inhibited the biosynthesis of thromboxane A2, leading to antiplatelet effect, but may increase the risk of bleeding [12]. Currently, hawthorn is widely used in cardiovascular diseases, especially arrhythmia [22], congestive heart failure [23–26], and hypertension [27]. Studies demonstrated that the biochemical mechanism of antiventricular arrhythmia is prolonging the APD through blocking the delayed (IK) and inward (IK1) rectifier potassium currents [22]. However, Long et al. [28] preferred that hawthorn was consistent with the effects profile of phosphodiesterase-3 (PDE3) inhibitors, which was different from Muller's opinion [22]. Hawthorn extract LI 132 (Faros® 300, CRA) was further found to increase cardiac contractility with prolonging the ERP, thus avoiding arrhythmogenic potential [26]. In addition, the other extract, WS 1442, would improve exercise capacity and then decrease mortality in heart failure patients [24, 25]. Furthermore, it was indicated that the epicatechin and hyperoside in the hawthorn fruit tincture [29] and the fluid extract of hawthorn had antioxidant activity [30]. From the above results, we find hawthorn extracts have the characteristics of antiplatelet aggregation, rhythm control, antioxidant activity, and management of the primary disease like heart failure, which makes it possible agent for AF treatment (Table 1). Nevertheless, the identity of the cardioactive constituent is still uncertain [31]. Therefore, corresponding experimental and clinical researches targeting hawthorn on AF are needed and deserved.

### 2.4. *Corydalis turtschaninovii* Besser

*Corydalis turtschaninovii* Besser (1834) is a medicinally important species of* Corydalis*, which comes from the family Papaveraceae. Its dried tubers, also known as Yanhusuo in China, can invigorate the circulation of blood and relieve pains, thus having been widely used in the treatment of cardiovascular diseases including arrhythmia [32]. Previous pharmacological studies reported that rotundium, an alkaloid of* Corydalis turtschaninovii* was antiarrhythmic by blocking the calcium channel in some animal experiments. And a later clinical research demonstrated that rotundium was an effective and safe medicine to treat AF, especially paroxysmal AF. The corresponding mechanism may be prolonging the ERP of atrial and atrioventricular node [33]. Except rotundium, there are four other alkaloids, d-corydaline, d-glaucine, protopine, and l-tetrahydrocolumbamine, isolated from the methanol extract of Chinese* Corydalis* tuber (CMe), which showed inhibitory action on blood platelet aggregation [34]. And the CMe was found to inhibit the decrease of blood platelets in disseminated intravascular coagulation (DIC) and inhibit pulmonary thromboembolism [34]. In addition, pseudocoptisine, a quaternary alkaloid with a benzylisoquinoline skeleton, was also extracted from the* Corydalis turtschaninovii* tubers and showed to have anti-inflammatory property. The potential molecular mechanism was reducing levels of the proinflammatory mediators, such as iNOS, COX-2, necrosis factor-alpha (TNF-alpha), and IL-6 through the inhibition of nuclear factor kappa B (NF-kappaB) activation via the suppression of ERK and p38 phosphorylation in RAW 264.7 cells [9]. Taken together, we can find that* Corydalis turtschaninovii* or its methanol extract has rhythm control or antiplatelet aggregation function. What is more, it can inhibit the inflammatory response that involves myocardial remodeling and thrombosis [35, 36]. Therefore,* Corydalis turtschaninovii* is a multifunctional agent (Table 1), which is worthy of in-depth researches targeting AF.

### 2.5. *Leonurus cardiaca* L. 

*Leonurus cardiaca* L. (1753), known as motherwort as well, is a herbaceous perennial plant in the mint family, Lamiaceae. It can be found worldwide, spreading largely due to its use as a herbal remedy. Motherwort with long history of use as a traditional herb in Asia is often used for uterine infection or other gynecological diseases in TCM, while the aqueous extracts from the aerial parts of* Leonurus cardiaca* have been used as a remedy against tachyarrhythmia and other cardiac disorders in Europe [37]. A research demonstrated that the primary and refined extracts of* Leonurus cardiaca* L. can inhibit the inward calcium (ICaL) and potassium (IKr) channels and lengthen Q-T, P-Q intervals and the activation time constant of I (f) in pacemaker cells, which lead to prolongation of cardiac cycle and activation recovery interval and APD in sinoatrial node cells and ventricular myocytes [37, 38]. Thus they decrease the blood pressure and heart rate and increase coronary blood flow and treat ventricular or sinus tachyarrhythmias [37]. In addition, motherwort also decreased blood viscosity and fibrinogen volume and increased the deformability of Rbc and antiplatelet aggregation effect [39]. Moreover, extracts from motherwort including polyphenolic compounds, mainly flavonoids (rutin) and derivatives of hydroxycinnamic acid, demonstrated antioxidant activity in several in vitro studies [10, 11, 29, 30, 40]. With the function of rate control, fibrinolysis, and antiplatelet aggregation effect and antioxidant activity which may be useful for addressing the primary disease (Table 1), motherwort or its extracts are promising and deserve further research in AF patients.

## 3. Traditional Chinese Patent Medicines

Traditional Chinese patent medicines are mainly comprised of Chinese herbal medicines or their extracts. With large numbers of clinical and experimental researches, many Chinese classical formulas are currently made into patent medicines for their efficacy, safety, and convenience. Therefore, more and more traditional Chinese patent medicines for specific diseases including AF are produced and used clinically.

### 3.1. Wenxin Granule (WXG)

Wenxin granule (WXG) is a Chinese medication, which contains* Codonopsis pilosula* Nannf.,* Polygonatum sibiricum* Red.,* Panax notoginseng*,* Nardostachys chinensis* Batal., and amber. WXG reportedly benefited patients with atrial arrhythmias and heart failure [41]. It is indicated to shorten conversion time, decrease the required dosage of amiodarone, and avoid the adverse reaction of long-term use of amiodarone, when combined with amiodarone [42]. A recent meta-analysis concluded that WXG alone or in combination with other antiarrhythmics decreased the recurrence of paroxysmal AF, with very few adverse effects [43]. The potential mechanism was demonstrated to be the inhibition of sodium channels (INa) selectivity in atrium, owing to more negative steady-state inactivation, less negative resting membrane potential, and shorter diastolic intervals in atrial versus ventricular cells at rapid activation rates [41, 44]. In addition, WXG produced prolongation of the ERP selectively in the atrial cardiomyocytes, without APD prolongation, thus lengthening the P-wave duration and preventing persistent AF [41]. This is a novel property, especially APD shortening and postrepolarization refractoriness in an atrial-selective manner, which is controversial to the well-known paradigm that efficacious atrial specific antiarrhythmic drugs have to significantly prolong APD and/or wave length [45]. What is more, another thought-provoking aspect was put forward by Kalifa and Avula [46]. They pointed out the fact that WXG significantly shortened APD90 in a manner that is not merely compatible with the late INa blockade, which usually produces only moderate APD shortening. So other ion currents besides INa are assumed in this performance. The ultrarapid delayed rectified potassium current (IKur) also presents in atria but not ventricles in human heart [47, 48]. So whether IKur is also participating in the action of WXG on AF deserves further discussion and research. Recently attentions have been already paid to developing selective inhibitors of the human atrial IKur or hKv1.5 channels [49], which may be a good news for AF patients. Additionally, WXG was found to lower AF inducibility after the ganglionic plexi (GP) ablation, without increasing the levels of atrial natriuretic peptide (ANP), tumour necrosis factor-alpha (TNF-*α*), interleukin- (IL-) 6, and expression of connexin 43 in atrial tissues, thus suppressing atrial substrate remodeling induced by GP ablation [50]. Although the current randomized controlled trials of WXG in treating AF (Table 2) are evaluated as low [43, 51], it surely shows certain treatment in different investigations and attracts people's attention to TCM.

### 3.2. ShenSongYangXin Capsule (SSYXC)

ShenSongYangXin capsule (SSYXC) is another Chinese medication which is made up of* Panax ginseng*,* Ophiopogon japonicus*,* Cornus officinalis* Sieb.,* Salvia miltiorrhiza* Bge.,* Ziziphus jujuba* Mill.,* Taxillus chinensis* Danser,* Paeonia lactiflora* Pall., ground beetle,* Nardostachys jatamansi*,* Coptis chinensis* Franch.,* Schisandra sphenanthera* Rehd., and fossilia ossia mastodi. SSYXC has been used for the treatment of tachyarrhythmias [52]. A randomized, double-blind, and controlled multicenter research has been conducted to find that SSYXC has efficacy in the treatment of paroxysmal AF, which is similar to propafenone [53]. It prolonged the APD significantly, with the potential mechanism of depressing the L-type calcium channel current (ICa-L), transient outward potassium current (Ito), and inward rectifier potassium current (IK1) in a concentration-dependent manner [54]. And the adverse reaction rate of this medication is indicated to be low [53]. Additionally, SSYXC is used for premature ventricular contractions and ventricular arrhythmias as well [55, 56], possibly by blocking multiple ion channels, namely, INa, ICaL, Ito, and IK1 [57, 58]. That is to say, it is a broad-spectrum antiarrhythmic drug, rather than the atrium-selective one. A meta-analysis [59] involving 22 trials and 2,347 paroxysmal AF (PAF) patients showed that although SSYXC appeared to improve P-wave dispersion (Pwd) and the frequency of PAF, the results included were inconsistent. Therefore, in order to further confirm the role of SSYXC (Table 2), more rigorous researches are needed to be done.

### 3.3. Shenmai Injection

Shenmai injection is derived from a Chinese classical formula which is an important component of TCM and has effective function of improving palpitation symptom. The injection is composed of ginseng saponins, radix ophiopogonis saponins, radix ophiopogonis flavonoids and traces of ginseng polysaccharides, and radix ophiopogonis polysaccharide as well. A current research showed that it could prolong ERP and convert AF into sinus rhythm, thus having synergistic effects with amiodarone [60] (Table 2). However, more reportedly studies are lacked to evaluate the function of shenmai injection on AF, and the potential mechanism is still unknown. Therefore, large numbers of experimental and clinical researches are urgently needed.

## 4. Nondrug Methods

TCM involves other therapeutic methods besides drugs, including acupuncture and Qigong that are illustrated to be effective in AF. Elderly people are the crowds in high risk of developing AF, with low level of liver and kidney condition; thus they easily get into drug intoxication. Therefore, nondrug methods are viable alternative for old patients and those with low tolerance for drugs.

### 4.1. Acupuncture

Acupuncture is an important and indispensable part of TCM. According to the traditional theory, many meridians and acupoints have antiarrhythmic effect, especially the Meridian of Minister of Heart that is associated with heart rate and blood flow. Neiguan spot is a spot in the area of this meridian, which locates in the middle of the forearm between the tendons [61]. It is an acupoint with high frequency of use and usually taken as the main spot for treating AF. Based on puncturing of the Neiguan, Shenmen, and Danzhong spots, a research probed the feasibility of acupuncture in conversion of paroxysmal AF and atrial flutter. The results showed that the rate of cardioversion was higher [62] and the duration to cardiovert [62, 63] was also shorter in patients treated with acupuncture than amiodarone. As to persistent AF, other studies have suggested that acupuncturing Neiguan, Shenmen, and Xinshu spots and so forth helped to decrease recurrences of persistent AF after cardioversion [63, 64], whose action was similar to amiodarone. In addition, the combination of acupuncture and Chinese patent medicine is perhaps a better choice for AF. A study indicated that, combined to simple Wenxin granule therapy in the treatment of paroxysmal AF, acupuncture combined with Wenxin granule had a better effect [65]. Therefore, acupuncture could be an effective nondrug and rhythm control tool in the management of these patients (Table 3). However, recent studies evaluating the effect of acupuncture are limited by small sample sizes and need to be validated in a larger population. And researches evaluating the mechanism of action have not yielded consistent results [66]; thus more experiments are ought to be conducted.

### 4.2. Qigong

Qigong is a special and unique part of TCM. Through physical exercises, it regulates the Qi of human body, which is a kind of material with important function, thus achieving health protection and treatment of diseases. Pippa et al. [67] confirmed that Qigong training was well tolerated and, compared with baseline, trained AF patients had better functional capacity and physical rehabilitation. In addition, Baduanjin, as one mind-body exercise of Qigong, could promote multisystem or organ functions (e.g., digestive and circulatory systems), increase immunity, make bodies relax, and improve mood and confidence of elderly populations [68]. Investigations further showed that Baduanjin was an effective therapy for hypertension [69] and dyslipidemia [70] and could reverse adverse left ventricular remodeling in post-myocardial infarction patients [71]. Additionally, it had beneficial effects on increasing antioxidant enzymes and reducing oxidative stress in middle-aged women by increasing superoxide dismutase and reducing malondialdehyde level [72]. Although Baduanjin was shown to be primary prevention of stroke in community old population with high risk factors [73], more rigorously designed RCTs about Baduanjin or Qigong and AF are still warranted. Therefore, Qigong is a promising and noninvasive method for management of primary diseases and risk factors for AF patients (Table 3), which need further mechanism researches.

### 4.3. Tai Chi Chuan (TCC)

Tai Chi Chuan (TCC) is also known as shadow boxing. It is another way of physical exercise that is a combination of martial art and the regulation of Qi in the theory of TCM. It integrates the breath, mind, and physical activity, thus making exercisers achieve greater awareness and a sense of inner peace. TCC has been popularly accepted and practised as a health care approach and an alternative method for the reduction of symptoms and risk factors in cardiovascular diseases (Table 3). A recent research further indicated its efficacy in the improvement of cardiorespiratory function for old adults [74]. And a meta-analysis demonstrated that TCC lowered blood triglyceride level with a trend to decrease blood total cholesterol level [76]. What is more, as an aerobic exercise of moderate intensity, TCC could maintain better health and improve quality of life, thus reducing CVD risk factors [75]. In addition, TCC intervention could produce positive effects on patients with mental problems [77]. Therefore, TCC would be a good option for cardiovascular patients and serves as an adjunctive exercise modality to rehabilitation programs for patients with CHD or CHF [78]. However, we failed to find direct researches concerning TCC and AF, which has a scientific priority for future investigation.

## 5. Discussion 

TCMs showed in the above researches indicate that they can work on more than one principle of AF management simultaneously through multichannels. By means of regulating ion channels [7, 8, 22, 33, 37, 38, 41, 54], they act positive roles in rhythm or rate control. They also give a hand to the risk factors by vasodilation [7], anti-inflammatory effect [9, 19], antioxidant activity [10, 30, 72], and even resistance of myocardium remodeling [50, 71] and benefit some primary diseases such as heart failure [7, 26, 41], hypertension [27, 69], and dyslipidemia [70, 76]. In addition, antiplatelet aggregation [12, 34, 39] and fibrinolysis effect [39] are showed in some CHMs or bioactive ingredients. Therefore, TCMs have four significant characters that can be put forward here. To begin with, some medicines including* Saussurea involucrata*,* Corydalis turtschaninovii* Besser, and Wenxin granule can selectively work on the atrial myocytes with no effect on ventricular electrical parameters. It is just the one that the current strategy for suppressing AF is developing [48, 79] and the one that existing antiarrhythmic drugs lack. Next, the multichannels and multifunctions increase the therapeutic management of AF but reduce the side effects and the amount of drugs that only target one principle. Moreover, since exercise capacity [23], physical rehabilitation [67], and some mental problems [77] can be improved by TCMs, especially the nondrug methods, patients' life quality can be markedly enhanced. Finally, as AF is age-related and the old person has low tolerance for drugs because of declined function of liver and kidney, TCMs which have low adverse reaction [43, 53] will be a better choice for them. Nevertheless, two problems still exist in the current results, namely, the relatively insufficient and poor clinical studies and the limitation in anticoagulation.

In conclusion, despite suffering from lack of adequate mechanistic and clinical studies, TCM research in this area is exciting and may lead to the development of new drugs just like the discovery of amiodarone which is extracted from a plant named* Ammi visnaga*.

## Figures and Tables

**Table 1:** Application of single CHMs or bioactive ingredients in AF.

Original plant	Medicinal part	Bioactive ingredient	Methods of study	Result	Mechanism	Effect on the four principles of AF
*Berberis vulgaris* L.	Root	Berberine	Cellular study (rat and human atrial cells)	Vasodilation, positive ionotropic and negative chronotropic actions	Inhibition of Ito by binding to open state channels or shifting of the steady-state inactivation curve of Ito [7]	Rate and rhythm control management of primary disease and risk factors
	Root	Berberine	Animal study (rabbit)	Suppressing AF	Inhibiting Ito [7], prolonging ERP and APD [14]	Rhythm control
	Root	Berberine	Cellular study (guinea pig cardiac myocytes)	Multichannel ion blocker	Inhibiting KATP [15], IKV [16], IKCa [16], IK1, and IK [17]	/

*Saussurea involucrata* (Kar. & Kir.) Sch. Bip.	Flower [19, 21], root [20]	Sesquiterpene lactone fraction [20]	Animal study (rat)	Anti-inflammatory and analgesic effects [19–21]	Stabilization of lysosomal membranes and an antiproliferative effect [20], preventing accumulation of inflammatory cells [21]	Management of risk factors
	Flower	Acacetin	Animal study (dog)	Suppressing AF	Inhibition of Kach, Kur, and Ito as an atrial-selective agent, prolonging APD and ERP without prolonging the corrected QT interval [8]	Rhythm control

*Crataegus rhipidophylla* Gand.	Flower heads	Catechin and epicatechin	Cellular study	Antiplatelet aggregation	Inhibiting the biosynthesis of thromboxane A2 [12]	Antithrombotic therapy
	Fruit	Extract (containing flavonoid and procyanidin)	Cellular study (guinea pig ventricular myocytes)	Antiventricular arrhythmia	Prolonging the APD through blocking the delayed (IK) and inward (IK1) rectifier potassium currents [22]	/
	Fruit	LI 132 (Faros® 300, CRA) [26], WS 1442 [24, 25]	Animal study (Wistar rats heart) [23] and isolated hearts (guinea pig) [26], clinical research [24, 25]	Positive ionotropic action, improving exercise capacity and decreasing mortality in heart failure patients [23–26]	Increasing Ca (2+)-concentration intracellularly [23], increasing cardiac contractility with prolongation of the effective refractory period [26]	Management of primary disease
	Fruit	Epicatechin, hyperoside, and fluid extract	Cellular study (DPPH and ABTS techniques)	Antioxidant activity [29, 30]	/	Management of risk factors

*Corydalis turtschaninovii* Besser	Tuber	Rotundium	Clinical research (AF patients)	Suppressing AF	Prolonging the ERP of atrial and atrioventricular node [33]	Rhythm control
	Tuber	D-Corydaline, d-glaucine, protopine, and l-tetrahydrocolumbamine	Animal study (rat)	Antiplatelet aggregation [34]	/	Antithrombotic therapy
	Tuber	Pseudocoptisine	Cellular study (RAW 264.7 murine macrophage cells)	Anti-inflammatory	Reducing levels of iNOS, COX-2, necrosis factor-alpha (TNF-alpha), and IL-6 through the inhibition of nuclear factor kappa B (NF-kappaB) activation via the suppression of ERK and p38 phosphorylation [9]	Management of risk factors

*Leonurus cardiaca* L.	Aerial parts	Primary and refined extracts	Cellular and organ studies (rabbit, rat, and guinea pig)	Decreasing the blood pressure, heart rate and increasing coronary blood flow and treating ventricular or sinus tachyarrhythmias [37]	Inhibiting the inward calcium (ICaL) and potassium (IKr) channels, lengthening Q-T, P-Q intervals and the activation time constant of I (f) in pacemaker cells [37, 38]	Rate control and management of risk factors
	Aerial parts	/	Clinical research	Fibrinolysis effect and antiplatelet aggregation	Decreasing blood viscosity, fibrinogen volume and increasing the deformability of Rbc [39]	Antithrombotic therapy
	Aerial parts	Rutin and derivatives of hydroxycinnamic acid	Cellular study	Antioxidant activity [10, 11, 29, 30, 40]	/	Management of risk factors

**Table 2:** Application of Traditional Chinese patent medicines in AF.

Chinese patent medicine	Component	Methods of study	Result	Mechanism	Effect on the four principles of AF
Wenxin granule	*Codonopsis pilosula* Nannf., *polygonatum sibiricum* Red., *Panax notoginseng*, *Nardostachys chinensis* Batal., and amber	Clinical research (AF patients) [39] and cellular study (canine right cardiomyocytes) [38]	Shortening conversion time, decreasing the required dosage of amiodarone and avoiding the adverse reaction [39], prolongation of the ERP selectively in the atrial cardiomyocytes without APD prolongation, lengthening the P-wave duration, and preventing persistent AF [38]	Inhibition of INa selectivity in atrium [38, 41]	Rhythm control
		Animal study (dog)	Lowering AF inducibility after the ganglionic plexi (GP) ablation	Suppressing atrial substrate remodeling without increasing the levels of ANP, TNF-*α*, and IL-6 and expression of connexin 43 in atrial tissues [47]	Management of risk factors and rhythm control

Shen Song Yang Xin capsule	*Panax ginseng*, *Ophiopogon japonicus*, *Cornus officinalis* Sieb., *Salvia miltiorrhiza* Bge., *Ziziphus jujuba* Mill., *Taxillus chinensis* Danser, *Paeonia lactiflora* Pall., ground beetle, *Nardostachys jatamansi*, *Coptis chinensis* Franch., *Schisandra sphenanthera* Rehd., and fossilia ossis mastodi	Clinical research (paroxysmal AF patients)	Paroxysmal AF [53]	/	Rhythm control
		Cellular study (rabbit pulmonary vein cardiomyocytes)	Prolonging APD	Depressing the ICa-L, Ito, and IK1 [54]	Rhythm control
		Animal study (rat) [55], clinical research (premature ventricular contractions patients) [56], and cellular study (guinea pig ventricular myocytes) [57]	Treating premature ventricular contractions and ventricular arrhythmias [55, 56]	Blocking INa, ICaL, Ito, and IK1 [57, 58]	/

Shenmai injection	Ginseng saponins, radix ophiopogonis saponins, radix ophiopogonis flavonoids and traces of ginseng polysaccharides, and radix ophiopogonis polysaccharide	Clinical research	Converting AF into sinus rhythm	Prolonging ERP [60]	Rhythm control

**Table 3:** Application of nondrug methods in AF.

Name	Methods of study	Result	Effect on the four principles of AF
Acupuncture	Clinical research (paroxysmal AF and atrial flutter patients)	Increasing the rate of cardioversion [62], lowering the duration of conversion time [62, 63]	Rhythm control
	Clinical research (paroxysmal and persistent AF patients)	Decreasing recurrences of persistent AF after cardioversion [63, 64]	Rhythm control

Qigong (including Baduanjin)	Clinical research	Improving physical rehabilitation of AF [67]	/
	Clinical research	Reversing adverse left ventricular remodeling [71]	Management of risk factors
	Clinical research	Promoting multisystem or organ functions, increasing immunity, making bodies relax, and improving mood and confidence of elderly populations [68]	Management risk factors
	Clinical research	Lowering blood pressure [69]	Management of primary disease
	Clinical research	Modulating the blood lipid metabolism [70]	Management of primary disease
	Clinical research	Antioxidant activity and reducing oxidative stress [72]	Management of risk factors

Tai Chi Chuan	Clinical research	Improvement of cardiorespiratory function [74]	Management of risk factors
	Clinical research	Reducing CVD risk factors [75]	Management of risk factors
	A meta-analysis	Lowering blood triglyceride level [76]	Management of primary disease
	A literature search	Improvement in mental health [77]	Management of risk factors
